# Germline variants observed in pediatric cancer patients related to hereditary breast and ovarian cancer in adults

**DOI:** 10.1002/ijc.70097

**Published:** 2025-08-26

**Authors:** Katharina Daugs, Danielle Brandes, Layal Yasin, Ammarah Anwar, Jubayer Alam, Yash Prasad, Jil Bartrina y Manns, Melina Mescher, Ute Fischer, Arndt Borkhardt, Triantafyllia Brozou, Stefanie V. Junk

**Affiliations:** ^1^ Department of Pediatric Oncology, Hematology and Clinical Immunology, Medical Faculty Heinrich Heine University Düsseldorf Düsseldorf Germany

**Keywords:** cancer predisposition, childhood cancer, genotype‐driven approach, hereditary breast and ovarian cancer (HBOC)

## Abstract

Genetic predisposition is a major cause of cancer, yet little is known about the role of adult cancer predisposition syndromes (CPSs) in childhood cancers. Although extensively studied in adults, information about the impact of germline variants in genes associated with hereditary breast and ovarian cancer (HBOC) remains scarce in the pediatric context. To elucidate whether (likely) pathogenic variants (LP/PVs) in 25 selected HBOC‐related genes may contribute to cancer risk in children, we analyzed the spectrum of occurring germline variants. We assessed 372 children (median age at diagnosis 5.1 [0–22.2] years; 160 girls [43%]), including 212 (57%) with hematologic neoplasms, 71 (19%) with brain tumors, and 89 (24%) with various solid entities. Twenty‐seven of 372 patients (7%) carried LP/PVs in the candidate genes; for 12 of 27 (44%) no CPS was suspected prior to genotyping. LP/PV carriers were particularly at risk for second malignancies (SMN; 5/27 vs. 13/345; OR = 5.8; *p* = .0021); yet, LP/PVs in SMN‐developing patients resided exclusively in *TP53* (*n* = 3), *NBN* (*n* = 1), and *ATM* (*n* = 1). Burden testing of our single‐center cohort revealed considerable associations between monoallelic LP/PVs in five HBOC‐related genes (*TP53*, *CHEK2*, *ATM*, *NF1*, and *NBN*) and pediatric cancers compared to healthy adults (gnomAD v.3.1.1, non‐cancer dataset). Joint analyses adding 1120 individuals from a previous study Zhang et al. (2015) confirmed significant associations for *TP53*, *CHEK2*, *NF1*, *and MSH2*. Monoallelic LP/PVs in constrained HBOC‐related genes are significantly associated with pediatric cancers. However, particularly in clinically unexpected cases, detection of contributing LP/PVs by genotype‐driven approaches may improve patient outcomes by enabling risk‐adapted therapy and surveillance.

## What's New?

Investigating the spectrum of (likely) pathogenic germline variants (LP/PVs) in hereditary breast and ovarian cancer (HBOC) genes in pediatric malignancies by unselected genotyping identified 44% (12/27) of clinically unsuspected cases. Burden testing demonstrated considerable associations between monoallelic LP/PVs in five HBOC genes and pediatric cancers. Carriers are particularly at risk for second malignancies Complementing previous studies, our findings emphasize the benefit of systematic, genotype‐driven approaches and the relevance of HBOC‐related LP/PVs in pediatric cancers and surveillance.

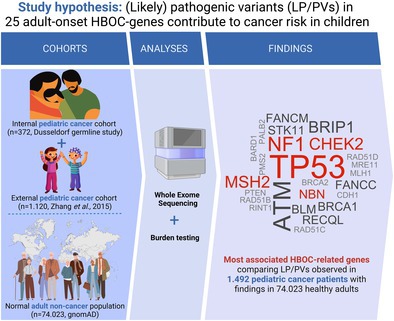

**Abbreviations**
AD: autosomal dominant
ALL: acute lymphoblastic leukemia
AML: acute myeloid leukemia
AR: autosomal recessive
AT: ataxia telangiectasia
BCP: B‐cell precursor
CI: confidence interval
CPG(s): cancer predisposition gene(s)
CPS(s): cancer predisposition syndrome(s)
DNA: deoxyribonucleic acid
gnomAD: genome aggregation database
HBOC: hereditary breast and ovarian cancer
HGNC: human gene nomenclature committee
HSCT: hematopoietic stem cell transplantation
LCH: Langerhans cell histiocytosis
LFS: Li‐Fraumeni syndrome
LOEUF/oe_lof_upper: loss‐of‐function observed/expected upper bound fraction/upper bound of 90% confidence interval for observed vs. expected ratio
LP: likely pathogenic
LPP [01‐27]: patient identifier for carrier of a (likely) pathogenic variant
NBS: Nijmegen breakage syndrome
PJS: Peutz‐Jeghers syndrome
pLoF: pathogenic loss‐of‐function
PV: pathogenic variant
SMN: second malignant neoplasm
WES: whole exome sequencing
*χ* ^2^
: chi‐squared (statistic)

## 1 INTRODUCTION

Up to 10% of children with pediatric malignancies have a cancer predisposition syndrome (CPS),^1^, ^2^ which in turn increases the risk for second malignant (primary) neoplasms (SMNs).^3^ Some cancer predisposition genes (CPGs) are well‐known to also promote pediatric cancer development, for example, *TP53*,^4^, ^5^ while the role of other adult‐onset CPGs, associated with hereditary breast and ovarian cancer (HBOC), remains poorly understood in pediatric malignancies. Germline alterations in *BRCA1* and *BRCA2* have been identified in 25% of adult‐onset HBOC cases.^6^ Female carriers of (likely) pathogenic variants (LP/PVs) in *BRCA1/2* are predisposed to ovarian cancer, while male carriers are at risk for prostate cancer, and both have an increased risk for breast and pancreatic cancer. Interestingly, the largest study to date including 1120 pediatric cancer patients identified the adult‐onset HBOC genes *TP53*, *BRCA2*, *NF1*, *ATM*, and *CHEK2* among the most frequently mutated genes^7^; two additional studies reported similar results.^8^, ^9^ However, no study focused on the mutational burden in this group of genes in pediatric patients.

Several HBOC‐susceptibility genes encode for tumor suppressors that contribute to genome stability/DNA‐repair pathways.^10^ Despite the clear association of these genes with adult‐onset HBOC, data on whether this familial predisposition also increases the risk of childhood cancer is inconsistent^11^, ^12^: for example, LP/PVs in *CHEK2* are associated with pediatric neuroblastoma and sarcoma^13^ and *MSH2* LP/PVs with Burkitt lymphoma.^14^ Furthermore, patients with certain CPSs, like Li‐Fraumeni syndrome (LFS), ataxia telangiectasia (AT), or the Nijmegen breakage syndrome (NBS), are particularly at risk for radiation‐induced SMNs and other chemotherapy‐related toxicities.^15^ LFS‐related *TP53* variants confer not only an increased lifetime risk for a variety of cancers, but also elevate the risk of SMNs by up to 49%.^16^ This raises the question of whether LP/PVs in other HBOC‐related CPGs are also associated with childhood cancers and whether more genetic testing and clinical screening are needed to avoid or reduce therapy‐related toxicities. Here we investigated the spectrum of LP/PVs in selected adult‐onset HBOC‐related genes in pediatric patients to determine their potential role in pediatric cancers and to enable the development of preventive measures regarding associated toxicities.

## 2 METHODS

All included patients were aged 1 to 22 at their first cancer diagnosis and enrolled in our “*Germline Mutations in Children with Cancer*” study between January 2015 and January 2023. This prospective study includes children and adolescents with newly diagnosed cancer, treated at the Department of Pediatric Oncology, Hematology and Clinical Immunology at the University Children's Hospital and their parents; for related details and whole exome sequencing (WES) analysis see Supporting Information Methods section and Brozou *et al*.^17^ Based on the literature^7^, ^8^, ^9^, ^10^ we selected 25 HBOC‐related candidate genes; for details see Table S1. Variant pathogenicity was assessed according to standard diagnostic guidelines and a standard gene‐based collapsing analysis for heterozygous LP/PVs was performed; details on both procedures are provided in the Supporting Information Methods section. Sequencing coverage and quality statistics for each LP/PV carrier are summarized in Table S2. Physical positions of determined variants are according to the human genome assembly GRCh38 (hg38).

## 3 RESULTS

Overall, 593 variants were identified in 297 of 372 analyzed pediatric cancer patients: 291 (49.1%) benign, 87 (14.7%) likely benign, 13 (2.2%) likely pathogenic, 15 (2.5%) pathogenic, and 187 (31.5%) variants of uncertain significance (VUS). Detailed patient information, including clinical phenotype and related LP/PVs, is presented in Table 1, Tables S3–S5, and Figure S1. We classified a total of 28 variants in 27 patients (7.3%) as LP/PVs. Only two patients carried more than one: one girl (LPP_20) carried two heterozygous LP/PVs in distinct genes (*CHEK2* and *NF1*) and one boy with typical clinical features of NBS (LPP_12) was homozygous for *NBN*(ENST00000265433.7):c.657_661del p.(Lys219Asnfs*16), rs587776650.

**TABLE 1:** Characteristics of 372 patients according to their carrier status regarding (likely) pathogenic variants in the 25 HBOC genes assessed.

		Patients with LP/PVs in a HBOC gene (*n* = 27), *n* (%)	Patients without LP/PVs in a HBOC gene (*n* = 345), *n* (%)	*p* ^a^, ^b^
Sex	Female	10 (37.0)	150 (43.5)	
	Male	17 (63.0)	195 (56.5)	.515
Age at initial diagnosis (years)	<3	9 (33.3)	94 (27.2)	
	≥3 < 9	7 (25.9)	137 (39.7)	
	≥9	11 (40.7)	113 (32.8)	.361
	Unknown	0 (0.0)	1 (0.3)	
First cancer entity	Leukemia	10 (37.0)	140 (40.6)	
	Lymphoma	5 (18.5)	43 (12.5)	
	Brain tumor	3 (11.1)	68 (19.7)	
	Solid tumor	8 (29.6)	81 (23.5)	
	Other neoplasm	1 (3.7)	13 (3.8)	.639^b^
Secondary events^c^	Second malignancy	5 (18.5)	13 (3.8)	
	Relapse	2 (7.4)	57 (16.5)	
	Progress	1 (3.7)	9 (2.6)	
	None	19 (70.4)	263 (76.2)	.014^b^
	Unknown	0 (0.0)	3 (0.9)	
Second malignant neoplasm	Yes, SMN	5 (18.5)	13 (3.8)	
	No	22 (81.5)	330 (95.7)	.006^b^
	Unknown	0 (0.0)	2 (0.6)	
Relapse of initial disease	Yes, Relapse	2 (7.4)	57 (16.5)	
	No	25 (92.6)	285 (82.6)	.280^b^
	Unknown	0 (0.0)	3 (0.9)	
Family history of cancer^d^	Yes	6 (22.2)	35 (10.1)	
	No	20 (74.1)	301 (87.2)	.098^b^
	Unknown	1 (3.7)	9 (2.6)	
CPS suspected (at least 1 DKG criterion met)^d^	Yes	14 (51.9)	122 (35.4)	
	No	12 (44.4)	214 (62.0)	.075
	Unknown	1 (3.7)	9 (2.6)	
Survival status	Alive	23 (85.2)	289 (83.8)	
	Deceased	1 (3.7)	50 (14.5)	.224^b^
	Unknown	3 (11.1)	6 (1.7)	

Abbreviations: CPS, cancer predisposition syndrome; DKG, German Cancer Society (“Deutsche Krebsgesellschaft”); HBOC, hereditary breast and ovarian cancer; LP/PVs, (likely) pathogenic variants; SMN, second malignant (primary) neoplasm.

^a^ *p*‐values resulting from the comparison of patients of the study cohort with at least one (likely) pathogenic variant (LP/PV) in one of the candidate genes compared to those without.

^b^ We applied the *χ* ^2^ statistic where possible; in cases where the criteria for the *χ* ^2^ statistic were not met, we performed the two‐sided Fisher–Freeman–Halton's exact test for the indicated variables with more than two categories and the Fisher's exact test for the others. Test results were considered significant at the .05 level.

^c^ Two patients experienced a relapse of the SMN.

^d^ For identification of potentially underlying cancer predisposition syndromes (CPSs) the questionnaire recommended by the DKG was applied; it is based on 6 criteria/categories, and according to Ripperger *et al*.,^1^ if at least one criterion is positive, genetic counseling is recommended. Family history was considered positive when ≥2 malignancies occurred in relatives before age 18, a parent or sibling had a current cancer or history of cancer before age 45, ≥2 first‐ or second‐degree relatives from the same parental lineage had cancer before age 45, or when the parents were consanguineous.

Comparing patients in our cohort with and without LP/PVs, there was no major difference in age at initial diagnosis, first cancer entity, or the male‐to‐female ratio (see Table 1). LP/PV carriers had a significantly higher rate of SMN (18.5% vs. 3.8%, *p* = .006); while fewer relapses were observed (7.4% vs. 16.5%, *p* = .280). Considering three‐generation pedigrees, six of 27 patients (22.2%) in our cohort with LP/PVs had a family history of cancer, compared with 10.1% of patients without. Overall, 135 of 372 patients (36.0%) were suspected to have a CPS based on their clinical phenotype, and 14 of 135 (10.4%) had at least one HBOC‐related LP/PV. Yet, 12 of 27 (44.4%) LP/PV carriers were not suspected of having CPSs prior to genotyping.

**FIGURE 1:**
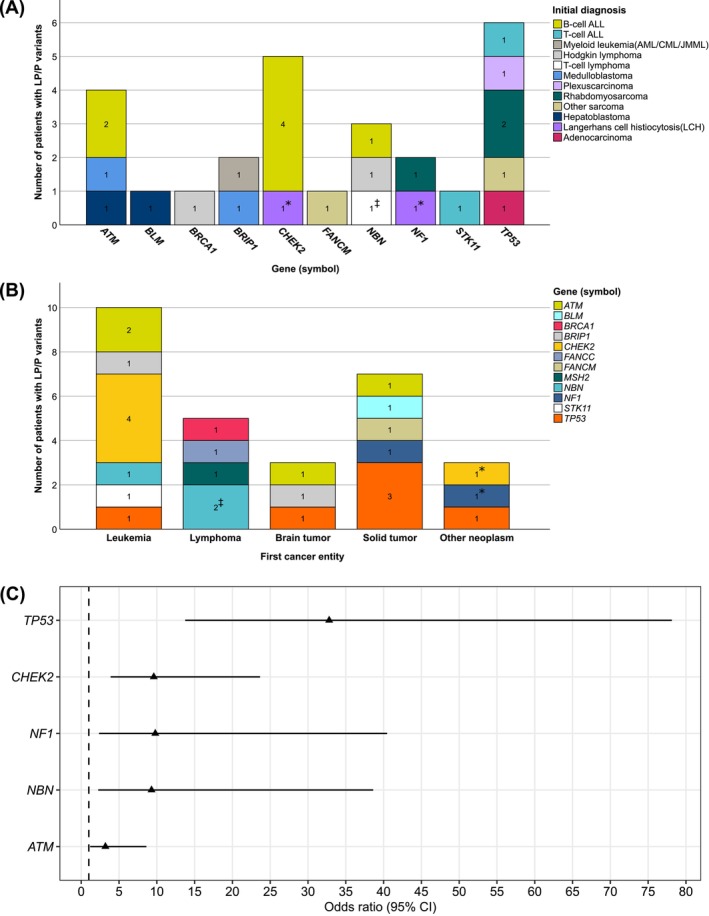
Patients with (likely) pathogenic (LP/P) variants per gene according to (A) their first cancer diagnosis; and (B) *vice versa* according to the initial cancer entity category. One patient with a Langerhans cell histiocytosis (LPP_20) carried two distinct heterozygous LP variants: one in *CHEK2* and one in *NF1*(*); a second patient with a T‐cell lymphoma (LPP_12) carried a homozygous *NBN* variant (ⱡ) and was excluded from monoallelic burden testing. Panel (C) shows the burden test results by gene for all patients of our internal cohort (*n* = 372), regardless of the initial entity and compared to the Genome Aggregation Database non‐cancer population (gnomAD non‐cancer, version 3.1.1). Statistically significant burden test results were obtained for heterozygous LP/PVs in *TP53*, *ATM*, *CHEK2*, *NBN*, and *NF1*.

In total, we determined LP/PVs in 12 of the 25 genes (see Figure 1A,B): six of 28 (21.4%) in *TP53*, five (17.9%) in *CHEK2*, and four (14.3%) in *ATM*. A further 13 LP/PVs were detected in *NBN* (*n* = 3), *BRIP1* (*n* = 2), *NF1* (n = 2), *BRCA1* (*n* = 1), *BLM* (*n* = 1), *FANCC* (*n* = 1), *FANCM* (*n* = 1), *MSH2* (*n* = 1), and *STK11* (*n* = 1). Regarding first cancer entities, leukemias were the most frequent among LP/PV carriers (37.0%), followed by solid tumors (29.6%) and lymphomas (18.5%). All LP/PVs determined in *ATM*, *NBN*, *BRIP1*, *STK11*, *MSH2*, *FANCM*, *BLM*, and *FANCC* were pathogenic loss‐of‐function variants (pLoF); for details, see Table 2. Besides pLoF, some individuals carried missense LP/PVs in *TP53* (*n* = 2), *CHEK2* (*n* = 3), and *BRCA1* (*n* = 1), and finally, one individual carried a truncating LP/PV in the intronic splice region of *NF1*.

**TABLE 2:** Burden test results by gene.

Gene^a^	Healthy adults, +LP/PV^b^	Healthy adults, −LP/PV^b^	Pediatric cases, +LP/PV^c^	Pediatric cases, −LP/PV^c^	OR (95% CI)^d^	*p* ^d^	n pLoF, *n* (%)^e^	LOEUF^f^
*TP53* ^g^	37	73,986	6	366	32.8 (13.8–78.1)	7.83 × 10^−8^	4 (66.7)	0.469
*CHEK2*	105	73,918	5	367	9.6 (3.9–23.7)	2.42 × 10^−4^	2 (40.0)	1.522
*ATM*	251	73,772	4	368	3.2 (1.2–8.6)	.040	4 (100.0)	0.710
*NF1* ^g^	41	73,982	2	370	9.8 (2.4–40.5)	.020	1 (50.0)	0.285
*NBN*	43	73,980	2	369	9.4 (2.3–38.7)	.021	2 (100.0)	1.010
*BRIP1*	95	73,928	2	370	4.2 (1.0–17.1)	.085	2 (100.0)	0.786
*STK11* ^g^	1	74,022	1	371	199.5 (12.5–3195.8)	.010	1 (100.0)	0.245
*MSH2*	14	74,009	1	371	14.2 (1.9–108.6)	.072	1 (100.0)	0.334
*FANCM*	91	73,932	1	371	2.2 (0.3–15.8)	.370	1 (100.0)	0.593
*BRCA1*	103	73,920	1	371	1.9 (0.3–13.9)	.406	0 (0.0)	0.920
*BLM* ^g^	118	73,905	1	371	1.7 (0.2–12.1)	.450	1 (100.0)	0.751
*FANCC*	151	73,872	1	371	1.3 (0.2–9.4)	.534	1 (100.0)	1.043

Abbreviations: 95% CI, 95% confidence intervals; HBOC, hereditary breast and ovarian cancer; LP/PVs, (likely) pathogenic variants; OR, odds ratio; pLoF, pathogenic loss‐of‐function variants.

^a^ Approved gene symbols, according to the Human Gene Nomenclature Committee (HGNC).

^b^ We included all available healthy adults from the Genome Aggregation Database (gnomAD) non‐cancer set version 3.1.1 as controls (healthy adults, *n* = 74,023) in our burden testing analyses.

^c^ For burden testing, we compared the number of LP/PVs per gene and per patient in our study cohort (pediatric cases) with the frequencies in the healthy adult control population. We included 372 individuals of our cohort with heterozygous variants in either *TP53*, *NF1*, *MSH2*, *FANCM*, *FANCC*, *CHEK2*, *BRIP1*, *BRCA1*, *BLM*, *ATM*, and *STK11*; for *NBN* analysis one individual (LPP_12) with a homozygous *NBN* variant was excluded (for details see Table S3).

^d^ For gene‐based burden testing, we performed logistic regression analyses to determine the odds ratios (OR) and 95% confidence intervals (95% CI); *p*‐values (*p*) were calculated using the two‐sided Fisher's exact test. Results were considered to be of relevance for *p*‐values <.05.

^e^ The number of loss‐of‐function variants among the identified LP/PVs per gene in patients of our cohort; variants were considered pLoF if the variant ontology was frame‐shift, nonsense, or splice donor/acceptor variants. In addition to pLoF, we identified six missense variants in the genes *TP53* (*n* = 2), *CHEK2* (*n* = 3), *BRCA1* (*n* = 1) and one intronic splice region variant in *NF1* as LP/PVs according to diagnostic variant classification standards.

^f^ As a metric for evolutionary pressure, mutational constraint scores are given to indicate the tolerance of a gene regarding pLoF variants in the normal (adult) population^22^, ^25^: that is, upper bound of 90% confidence interval for observed vs. expected ratio for pLoF variants (LOEUF/oe_lof_upper, gnomAD v.2.1.1); values <.35 are considered to be constrained.

^g^ Diseases related to these genes exclusively follow an autosomal dominant inheritance mode.

Distinct monoallelic *ATM* LP/PVs were observed in two B‐cell precursor acute lymphoblastic leukemia (BCP‐ALL) patients (LPP_02 and LPP_03) and two in individuals with medulloblastoma and hepatoblastoma, respectively (LPP_01 and LPP_04). We detected five *CHEK2* LP/PVs: four in BCP‐ALL patients and one in a patient with Langerhans cell histiocytosis (LCH, LPP_20). Notably, among the six BCP‐ALL patients with *ATM* or *CHEK2* LP/PVs, one relapsed (LPP_17), and three had other cases of leukemia in their immediate families; for details, see Table S3. Out of six children with *TP53* LP/PVs, five had a family history of cancer, including two with relatives who also experienced pediatric cancer. The LFS‐related variant TP53(ENST00000269305.8):c.586C>T p.(Arg196*) was detected twice: First, *de novo* in a boy, LPP_07, with a negative family history of cancer who developed a T‐cell ALL at age 8.7. Within 14 months, a lineage switch to hyperdiploid BCP‐ALL occurred, followed by allogeneic hematopoietic stem cell transplantation (HSCT) four months later and a BCP‐ALL relapse six months after HSCT. Due to severe neurotoxicity during conditioning therapy with fludarabine (initially muscle atrophy, followed by right‐side hemiparesis progressing to tetraparesis) and the need for daily transfusions, no further therapy was administered. Unfortunately, he died one month later. Second, we observed p.(Arg196*) in patient LPP_10, who underwent an adenocarcinoma removal at age 1.8 and two subsequent cancers: BCP‐ALL at 4.8 years and acute myeloid leukemia (AML) during the ALL maintenance therapy. Diagnosed with LFS, she received a personalized chemotherapy regimen with reduced doses of cytarabine and gemtuzumab. After stem cell rejection from her mother's haploidentical HSCT, she achieved remission following HSCT from her maternal grandmother seven months ago and is currently in good clinical condition. Besides the homozygous carrier of *NBN*(ENST00000265433.7):c.657_661del p.(Lys219Asnfs*16), rs587776650, we identified two patients with hematologic neoplasms who were heterozygous for this known founder mutation. Both achieved remission without experiencing secondary events. Patient LPP_27 carried the pathogenic variant *STK11*(ENST00000326873.11):c.179dup p.(Tyr60*). Consistently, this boy presented with congenital signs of Peutz‐Jeghers syndrome (PJS) and a T‐cell ALL, along with a family history of cancer; his mother and maternal grandfather also have PJS. One 11.4‐year‐old girl, LPP_20, diagnosed with LCH, carried two LP/PVs: *NF1*(ENST00000358273.8):c.4137dup p.(Ala1380Serfs*15) and *CHEK2*(ENST00000382580.6):c.1165C>T p.(Arg389Cys). She had no typical neurofibromatosis type 1‐related symptoms, but a positive family history of late‐onset cancers, including breast cancer and acute and chronic myeloid leukemias.

Subsequently, we compared the burden of monoallelic LP/PVs per individual and per gene in our patients with the findings observed in healthy adults (*n* = 74,023, gnomAD non‐cancer dataset, version 3.1.1). The resulting odds ratios (OR) and related statistical measures are shown in Table 2, Figure 1C, and in Figure S2. Burden testing revealed significantly higher ORs for patients in our cohort for heterozygous LP/PVs in *TP53*, *NF1*, *NBN*, *CHEK2*, and *ATM*, suggesting that mutations in these five genes were associated with childhood cancers. By contrast, the obtained associations for LP/PVs in *BRIP1*, *MSH2, STK11*, *BLM*, *BRCA1*, *FANCC*, and *FANCM* were not considered statistically significant. To augment the statistical power, we included the genetic information of 1120 individuals published by Zhang *et al* 2015^7^; see Table S6 and Figure S3 for details. This joint burden testing confirmed the strong associations of *TP53*, *NF1*, *and CHEK2* with pediatric cancers. In contrast to our initial approach, we determined that *MSH2* LP/PVs carriers are also particularly at risk of developing pediatric cancer (OR = 7.1, 95% CI = 1.6–31.2, *p* = .0390). No reliable associations were obtained for the remaining candidate genes.

## 4 DISCUSSION

Previous studies on pediatric cancer patients identified 8 to 10% with germline mutations in CPGs.^1^, ^7^, ^8^ Only a few, however, focused on CPS involvement^1^, ^18^, ^19^ or pediatric‐adult phenotype discrepancies.^7^, ^20^ Similar to studies on general CPGs,^7^, ^8^, ^18^, ^19^ we observed 7.3% of patients with HBOC‐related LP/PVs. Despite stringent clinical screening, according to standardized clinical procedures,^1^ 44% of our LP/PV carriers were not suspected of having a CPS prior to genotyping, contrasting with a recent study reporting only 4% of pediatric cases diagnosed based solely on genotyping.^19^ Regarding general CPGs, approximately 50% of patients with germline LP/PVs have a positive family history.^7^, ^19^, ^21^ Focusing on HBOC‐related genes, we observed only 22% with such a history, which may either indicate a lower rate for those genes or an underrepresentation in our cohort. Long‐term follow‐up will be required to clarify. Some of our patients exhibited clinical features typical of the HBOC genes involved, for example, the *TP53* LP/PV carriers with rhabdomyosarcomas (LPP_05 and LPP_08). Besides patient LPP_05, who subsequently had two distinct osteosarcomas, two other patients with truncating PVs in *TP53* also developed SMNs (LPP_07 and LPP_10); one of them died thereafter. Consistent with previous observations in childhood cancer suvivors,^16^ in our cohort 3 of 6 (50%) *TP53* LP/PV carriers developed SMNs. The same holds true for two additional patients with LP/PVs involving *NBN* (*n* = 1) and *ATM* (n = 1), which was not surprising, since *TP53*, *NBN*, and *ATM* mutations are associated with extensive treatment‐related toxicities, including SMN development.^10^, ^15^ Overall, LP/PV carriers in our cohort had a 5.8‐fold increased SMN risk (95% CI = 1.89–17.75, *p* = .0021); notably, all of them with LP/PVs in genes that are well‐known for early‐onset cancers and SMN development when functionally impaired. While *TP53*‐related LFS is inherited autosomal dominantly, AT and NBS are autosomal recessive, requiring biallelic mutational impairment. Nonetheless, we report four children with monoallelic truncating *ATM* variants without AT‐related features, one of whom also experienced secondary events. Similarly, we identified two individuals with heterozygous *NBN* variants who developed hematologic neoplasms without further NBS‐related symptoms, suggesting that monoallelic variants in AR genes may also contribute to pediatric cancer risk.

Recent investigations revealed that highly constrained genes are intolerant to functional variants, making them more likely to be pathogenic.^22^ So possibly reflecting evolutionary pressures, our candidate genes *NF1, MSH2*, and *STK11* are highly constrained; *TP53* and *ATM* are moderately constrained, and for example, *CHEK2 and NBN* are less constrained. Interestingly, and close to our findings, in that study^22^ the HBOC‐related *TP53*, *BRCA2*, *NF1*, and *PMS2* were among the most associated genes regarding autosomal dominant (AD) pediatric CPSs. Biallelic impairment is needed for the manifestation of autosomal recessive diseases, while monoallelic *TP53*, *CHEK2*, and *NF1* pLoF are sufficient to promote cancer development. Consistently, *NF1*, along with *TP53* and *CHEK2*, was among the top five associated genes in the initial burden testing of our single‐center cohort. Unexpectedly, monoallelic LP/PVs in *ATM* and *NBN* also reached significant levels, while other promising candidates, for example, *MSH2* or *BRIP1*, failed. By including genetic information from an additional 1120 patients previously published by Zhang *et al*,^7^ we aimed to overcome existing statistical limitations. In this joint approach, we determined significant associations between LP/PVs in *NF1* and *MSH2* and pediatric cancers, while no significant results were obtained for the less constrained genes, for example, *ATM* or *BRCA1*. Surprisingly, we observed an almost significant association for unconstrained *NBN*. Since BCP‐ALL patients with germline *NBN* LP/PVs were shown to have similar outcomes compared to non‐carriers^23^; this may suggest that the mutational impact is unlikely to be so fatal that variants could not be passed on. However, highly constrained *NF1* and *MSH2* are frequently mutated in pediatric cancer patients.^7^, ^9^ *MSH2* is particularly noteworthy in our context: a meta‐analysis restricted to nine DNA‐repair genes reported *MSH2* as the only one overall consistently associated with childhood cancer risk. Furthermore, the association of the highly penetrant AD and the AR *MSH2* phenotypes was correlated with its intolerance to pLoF variants.^22^

Overall, our results confirm that well‐established CPGs such as *TP53* or *ATM* are frequently affected by heterozygous LP/PVs, underlining their important role in pediatric cancers. Intriguingly, we observed an accumulation of cases with monoallelic *ATM* and *NBN* LP/PVs, genes which are typically associated with AR syndromes, suggesting a potentially underexplored role related to pediatric cancer predisposition. The strong associations obtained for LP/PVs in DNA‐repair genes may reflect the importance of these processes in pediatric oncogenesis. Our findings support the hypothesis that highly constrained genes, like *NF1* and *MSH2*, are more likely to contribute to pediatric cancer development when mutated. By focusing on only the 25 most relevant HBOC‐related genes and omitting other related genes like *MSH6*, *ERCC2*, or *CDKN2A* ^7^, ^22^, ^24^ our study may be limited. Furthermore, due to technical limitations, copy‐number variations were not considered; the lack of available tumor material precluded the investigation of second‐hit variants, and finally, varying follow‐up times may lead to an incomplete picture regarding SMN development. Yet, our results emphasize the necessity of systematic genotype‐driven approaches to identifying unsuspected cases, particularly in the absence of clinical risk factors. Thereby‐determined (monoallelic) HBOC‐related LP/PVs may improve pediatric patient outcomes by enabling enhanced preventive measures to reduce toxic side effects and early detection of SMNs; moreover, it may also help to identify relatives at risk.

## AUTHOR CONTRIBUTIONS

**Katharina Daugs:** Investigation; writing – original draft; data curation. **Danielle Brandes:** Validation, Writing – review & editing, investigation. **Layal Yasin:** Visualization; formal analysis; software; supervision. **Ammarah Anwar:** Visualization; data curation; software. **Jubayer Alam:** Formal analysis; visualization; software. **Yash Prasad:** Data curation; formal analysis. **Jil Bartrina y Manns:** Data curation; investigation. **Melina Mescher:** Visualization. **Ute Fischer:** Funding acquisition; writing – review and editing; project administration; supervision; resources. **Arndt Borkhardt:** Supervision; project administration; writing – review and editing; funding acquisition; conceptualization; resources; methodology. **Triantafyllia Brozou:** Conceptualization; funding acquisition; writing – review and editing; data curation; resources; project administration. **Stefanie V. Junk:** Project administration; writing – original draft; investigation; conceptualization; writing – review and editing; formal analysis; supervision; validation; methodology.

## CONFLICT OF INTEREST STATEMENT

The authors have no conflicts of interest to declare regarding the present study. The data presented here have not been previously presented and have not been previously submitted for publication in any other journal.

## ETHICS STATEMENT

The study was approved by the Institutional Ethics Committee of Heinrich Heine University, Düsseldorf, Germany (study number 4886 study registration number 2014112933) in 2015, and informed consent was obtained from patients and/or their guardians.

## Supporting information


**Data S1.** Supporting information.

**Data S2.** Supporting information.

## Data Availability

All relevant information and data generated or analyzed during this study are included in this published article and its Supporting Information files. The WES data of the 27 LPP cases identified with LP/PVs in this study is available in EGA under accession number EGAD50000001561. Additional data supporting the findings of this study are available from the corresponding author upon request.
