# Supplementary material for: Germline variants observed in pediatric cancer patients related to hereditary breast and ovarian cancer in adults
Source: Int J Cancer. 2025 Aug 26;157(12):2447–54. doi: 10.1002/ijc.70097 (PMC12541558; doi:10.1002/ijc.70097)
Supplement: Supplementary file 1 — Data S1. Supporting information. [file IJC-157-2447-s002.pdf]

Supplementary Information to

Germline variants observed in pediatric cancer patients related to hereditary breast and ovarian cancer in adults

Katharina Daugs, Danielle Brandes, Layal Yasin, Ammarah Anwar, Jubayer Alam, Yash Prasad, Jil Bartrina y Manns, Melina Mescher, Ute Fischer, Arndt Borkhardt, Triantafyllia Brozou and Stefanie V. Junk

Supplementary Appendix Content

Supplementary Methods

Supplementary Tables

Suppl. Table 1. Details on selected candidate genes.

Suppl. Table 2. Sequencing coverage and quality statistics for each LP/PVs carrier sample included in this study (n=27) (see additional supplementary excel file).

Suppl. Table 3. Clinical information on patients with (likely) pathogenic variants in our study population.

Suppl. Table 4. Details on determined (likely) pathogenic variants and pathogenicity classification.

Suppl. Table 5. Information on 187 determined variants of uncertain significance (VUS) in 372 analyzed pediatric cancer patients (see additional supplementary excel file).

Suppl. Table 6. Joint burden testing results by gene.

Supplementary Figures

Suppl. Figure 1. Oncoprint visualization of all 28 determined (likely) pathogenic (LP/P) variants by gene and the categorized first cancer entity of the carrier.

Suppl. Figure 2. Non-significant burden test results by gene (internal cohort, n=372).

Suppl. Figure 3. Joint burden testing results (n=1492).

Supplementary References

Supplementary Methods

Study concept

The complete outline of the study was previously described[1]. Briefly, after obtaining an informed consent, from each child and/or the parents, an experienced pediatrician collected demographic data and family cancer history for three generations and performed clinical examinations for congenital anomalies. Germline DNA from the children was analyzed by WES. When possible, both parents were included to identify potential consanguinity and de novo mutations. Detected LP/PVs were correlated with cancer types and patient histories.

Whole exome sequencing with focus on 25 candidate genes

We performed (paired-end) whole exome sequencing (WES) on either a NextSeq550 or a NextSeq2000 (2 × 150 bp) sequencing platform (Illumina GmbH, Berlin, Germany) using a SureSelect Human All Exon V5+UTR kit, All Exon V7 or All Exon V8 (Agilent, Waldbronn, Germany); annotation and final filtering on the 25 candidate genes was carried out using an in-house developed bioinformatic pipeline based on GATK best practice recommendations (version 4.2.0.0) and Ensembl Variant Effect Predictor (VEP, release 103,GRCh38)[2]. In sequencing analyses of the exons (±70 base pairs), minimum depth was 100x. Copy number variations were not analyzed in this WES-based approach. Sequencing coverage and quality statistics for each included sample are summarized in Suppl. Table 2.

Variant pathogenicity was classified according current standard diagnostic guidelines, i.e., the *American College of Medical Genetics and Genomics* (ACMG) criteria[3] and additional gene-specific refinements, when recommended by the *Clinical Genome Resource* (ClinGen)[4] expert panel consortium. Classification was performed independently by two investigators and then extensively discussed in the context of the available literature and database information (gnomAD v.3.1.2, ClinVar, ClinGen, LOVD3, INSiGHT, BRCA Exchange, NCI-*TP53* database (formerly IARC) etc.). Whenever available curated variant classifications based on the reviews of ClinGen expert panels were included. All classifications presented here are the result of this evaluation process.

Statistical analyses and burden testing

The statistical analysis and subsequent graphical representation of the data were performed using SPSS Statistics (IBM Deutschland GmbH, Ehningen, Germany) or R (4.0.5) and Rstudio (1.0.153).

We performed a gene-based burden test, using binary logistic regressions and a 2-sided Fisher's exact test: For each gene we compared the number of individuals with heterozygous/monoallelic LP/PVs in our cohort with the corresponding data available from the gnomAD non-cancer dataset; variants flagged by the gnomAD committee due to quality issues were excluded from the analysis. *P*-values <0.05 were considered to be of relevance. Patient 12, the only individual in our cohort with a biallelic variant, homozygously carried the known founder mutation *NBN*(ENST00000265433.7):c.657_661del p.(Lys219Asnfs*16) and was therefore excluded from subsequent burden testing analysis for *NBN*.

Supplementary Table 1. Details on selected candidate genes.

Gene[a]	ClinGen refinement[b]	Gene ID[c]	Associated phenotype	Inheritance[d]
CHEK2[5,6]	-	11200	Hereditary breast and ovarian cancer, hereditary SNVs are associated with hematologic neoplasms (association to Li-Fraumeni syndrome is outdated)[7]	AD
CDH1	GN007	999	Familial diffuse gastric cancer with or without cleft lip and/or palate	AD
NF1[3]	-	4763	Neurofibromatosis type 1	AD
PTEN	GN003	5728	Cowden syndrome 1	AD
TP53	GN009	7157	Li-Fraumeni syndrome	AD
BRCA1	GN092	672	Breast and ovarian cancer (AD) / Fanconi anemia (AR)	AD / AR
BRCA2	GN097	675	Familial breast and ovarian cancer (AD), medulloblastoma, glioblastoma, Wilms tumor (AD)/ Fanconi anemia(AR)	AD / AR
FANCC[3]	-	3584	Fanconi anemia	AR
FANCM[3]	-	2175	Fanconi anemia	AR
MSH2[8,ƚ]	-	4436	Lynch (AD) / Constitutional mismatch repair deficiency syndrome(AR)	AD / AR
PALB2	GN077	79728	Fanconi anemia	AD
PMS2[8,ƚ]	-	5395	Lynch / Constitutional mismatch repair deficiency syndrome	(AD) / AR
ATM	GN020	472	*Ataxia telangiectasia (AR)* / susceptibility to breast cancer(AD/SMu)	AR / AD(SMu)
BLM[3]	-	641	Bloom syndrome	AR
NBN[3]	-	4683	Nijmegen breakage syndrome	AR
RECQL[3]	-	9948	Breast cancer susceptibility	AD / AR / ?
STK11[3]	-	6794	Peutz-Jeghers syndrome	AD
BRIP1[5]	-	83990	Familial breast and ovarian cancer(AD)/Fanconi anemia(AR)	AR
MLH1[8,ƚ]	-	4292	Lynch / Constitutional missmatch repair deficiency syndrome	AR
MRE11[3]	-	7230	Ataxia-telangiectasia-like disorder 1	AR
RAD51B[3]	-	5890	Familial ovarian cancer/hereditary breast carcinoma	AR
RAD51C[5]	-	5889	Fanconi anemia/familial ovarian cancer/hereditary breast carcinoma	AR
RAD51D[5]	-	5892	Fanconi anemia/familial ovarian cancer/hereditary breast carcinoma	AD / AR
BARD1[3]	-	580	Hereditary breast carcinoma/hereditary nonpolyposis colon cancer/familial ovarian cancer	AD / SMu
RINT1[3]	-	60561	Breast cancer / Lynch syndrome-spectrum cancers	AR

Abbreviations: Acute lymphoblastic leukemia (ALL); acute myeloid leukemia (AML); myelodysplastic syndrome (MDS); Non-B-cell non-Hodgkin lymphoma (NB-NHL); non-Hodgkin lymphoma (NHL); second malignant neoplasm (SMN), single nucleotide variants (SNV).

[a] Approved gene symbols, according to the human gene nomenclature committee (HGNC). All variants determined in this project were assessed according to standard variant interpretation guidelines as indicated above for each gene (see indicated references). Variants in the mismatch repair genes were classified according to the most recent version of the "InSiGHT Variant Interpretation Committee MMR gene variant classification criteria" published in 2018 (see https://www.insight-group.org/criteria/;ƚ).

[b] Variants were classified according to the ACMG guidelines[3] and when available according to gene-specific refinements available via the ClinGen website. (https://cspec.genome.network/cspec/ui/svi/doc).

[c] Approved gene identifier (Entrez ID/GeneID; National Center for Biotechnology Information (NCBI)'s database for gene-specific information).

[d] Known inheritance modes: autosomal dominant (AD) and autosomal recessive (AR) or somatic mutation caused susceptibility (SMu); patients with a heterozygous deleterious mutation in a gene related to conditions/CPS with a known AR inheritance mode were considered to have a carrier status.

Supplementary Table 2. Sequencing coverage and quality statistics for each LP/PVs carrier sample included in this study (n=27) (for details see additional supplementary excel file).

Supplementary Table 3. Clinical information on patients with (likely) pathogenic variants in our study population.

LPP case	Sex	Age at diagnosis [y]	Diagnosis (first cancer)	Family history of cancer	Current status	Finding and molecular consequence	Variant(s) *de novo*
01	M	4.3	Medulloblastoma	-	Metastasis 12 y later; SMN (adenocarcinoma, 16 y later); detected T-cell defect and thrombocytopenia	*ATM*(ENST00000278616.8):c.5347_5350del p.(Glu1783Thrfs*9)	no
02	M	3.7	BCP-ALL; HHD karyotyp; *CDKN2A* Deletion	-	Remission	*ATM*(ENST00000278616.8):c 3880dup p.(Ile1294Asnfs*8)	no
03	M	3.0	Pro-B-ALL, (Nuc ish 3q34(ABL1x2), 22q11(BCRx2)[95/100], 11q23(MLLx2)[99/100])	Child: SGA, renal dysplasia left; GM (M): BC at age 57	Remission	*ATM*(ENST00000278616.8):c.1564_1565delp. (Glu522Ilefs*43)	no
04	F	0.6	Hepatoblastoma	-	Remission	*ATM*(ENST00000278616.8): c.8766dup p.(Val2923Cysfs*2)	no
05	M	1.7	Rhabdomyosarcoma	Child: status after IVF, eclampsia; GM (F): tumor death; U (F): childhood cancer	SMN (two distinct osteosarcomas at 9 and 13 y after initial diagnosis); now remission	*TP53*(ENST00000269305.8):c.97-6_129del p.?	no
06	M	0.6	Plexuscarcinoma	GM (F): BC at age 48; Uncle (F): childhood cancer at age 17	Relapse; now remission	*TP53*(ENST00000269305.8):c.733G>A p.(Gly245Ser)	no
07	M	8.5	TCP-ALL	-	SMN (BCP-ALL, 4 months later), followed by allogenic HSCT 4 months later; relapse of BCP-ALL at 6 months after allogenic HSCT; no further therapy and death 1 month later	*TP53*(ENST00000269305.8):c.586C>T p.(Arg196*)	yes
08	M	1.7	Embryonal rhabdomyosarcoma	GGP (F): gastric cancer and skin cancer; father carries the same *TP53* mutation	Remission	*TP53*(ENST00000269305.8):c.537T>G p.(His179Gln)	no
09	F	10.5	Osteosarcoma	GF (M): lung cancer (dead); GM (F): BC; GF (F): testicular cancer	Remission	*TP53*(ENST00000269305.8):c.919+1G>A p.?	no
10	F	4.8	Adenocarcinoma	Child: Li-Fraumeni syndrome	Two SMN (3 y later: BCP-ALL and at 5 y after initial diagnosis: AML followed by HSCT)	*TP53*(ENST00000269305.8):c.586C>T p.(Arg196*)	no
11	F	15.5	Hodgkin lymphoma	-	Remission	*NBN*(ENST00000265433.7):c.657_661del p.(Lys219Asnfs*16)	no
12	M	9.6	T-cell lymphoma	Child: Nijmegen breakage syndrome; prematurely born infant presenting with disproportionate hypotrophy, microcephaly, and recurrent respiratory infections	SMN (Burkitt lymphoma 5.7 years later) then remission, HSCT at age of 16.2	*NBN*(ENST00000265433.7):c.657_661del p.(Lys219Asnfs*16)[ǂ]	no
13	M	5.5	BCP-ALL (*ETV6::RUNX1*)	GM (M): endometrial cancer >age 50; GF (F): gastric cancer	Remission	*NBN*(ENST00000265433.7):c.657_661del p.(Lys219Asnfs*16)	unclear
14	M	12.8	AML t(8;21) (q22;q22) *RUNX1/RUNX1T1* positive; *NRAS* p.(Gly12Ala) in tumor	GF (M): prostate cancer at age 75 GF (F): lung cancer at age 65	Remission	*BRIP1*(ENST00000259008.6):c.2992_2995del p.(Lys998Glufs*60)	no

LPP case	Sex	Age at diagnosis [y]	Diagnosis (first cancer)	Family history of cancer	Current status	Finding and molecular consequence	Variant(s) *de novo*
15	F	12.7	Medulloblastoma classical WNT	Child: congenital ASD; GM (F): endometrial cancer; GF (F): melanoma; U (M): RMS (childhood cancer)	Remission	*BRIP1*(ENST00000259008.6):c.2400C>G p.(Tyr800*)	no
16	M	13.3	Burkitt lymphoma	-	Remission	*MSH2*(ENST00000233146.6):c.1386+1G>T p.?	no
17	M	9.4	BCP-ALL; HHD 53~56	-	Relapse; now Remission	*CHEK2*(ENST00000382580.6):c.1031del p.(Leu344Trpfs*3)	no
18	F	1.9	BCP-ALL; 46, XX,inc[4].nuc ishq34(ABL1x2), 22q11(BCRx2)[95/100]	Child: microcephaly; parents consanguine (cousins) GM (M): leukemia/lymphoma	Remission	*CHEK2*(ENST00000382580.6):c.628G>A p.(Gly210Arg)	no
19	M	12.2	BCP-ALL; 6, XY, -8, del(12)(p12), +mar[1]/46, XY[24]PAX5-SOX5-	Child: developmental delay, spherocytosis; GF (M): pancreatic cancer < age 45	Remission	*CHEK2*(ENST00000382580.6):c.573+1G>A p.?	no
20	F	11.4	Langerhans cell histiocytosis	GF (M): CML at age 59 GM (F): BC at age 67 GF (F): AML at age 70	Remission	*CHEK2*(ENST00000382580.6):c.1165C>T p.(Arg389Cys); *NF1*(ENST00000358273.8):c.4137dup p.(Ala1380Serfs*15)	no
21	F	2.7	BCP-ALL; *ETV6-NECAB3* fusion transcript	GF (M): leukemia at age 60 GM (F): skin cancer < age 45	Remission	*CHEK2*(ENST00000382580.6):c.1165C>T p.(Arg389Cys)	no
22	M	4.6	Embryonal rhabdomyosarcoma	Child : café au lait spots ; parents consanguine	Remission	*NF1*(ENST00000358273.8):c.6819+3del p.?	yes
23	F	17.4	Hodgkin lymphoma	F: lymphoma at age 30	Remission	*BRCA1*(ENST00000471181.7):c.5095C>T p.(Arg1699Trp)	unclear
24	M	0.5	DLBCL	Child: CMV-Pneumonia, EBV-Viremia	-	*FANCC*(ENST00000289081.8):c.37C>T p. (Gln13*)	no
25	F	16.6	Myofibroblastic sarcroma	GF (M): prostate cancer at age 65; GM (M): liver cancer, A (M): liver cancer > age 60	Progress	*FANCM*(ENST00000267430.10):c.4064del p.(Lys1355Argfs*14)	no
26	M	1.1	Hepatoblastoma	-	Remission	*BLM*(ENST00000355112.8):c.3558+1G>T p.?	no
27	M	3.0	TCP-ALL	Child: congenital hyperpigmented lips; PJS; M: PJS; GM (M): BC; GF (F): melanoma at age 75; GF (M): PJS	Remission	*STK11*(ENST00000326873.11):c179dup p.(Tyr60*)	no

Abbreviations: Patient identifier for carrier of a (likely) pathogenic variant (LPP); female (F); male (M); acute lymphoblastic leukemia (ALL); acute myeloid leukemia (AML); chronic myeloid leukemia (CML); B-cell precursor acute lymphoblastic leukemia (BCP-ALL); chronic myeloid leukemia (CML); T-cell precursor acute lymphoblastic leukemia (TCP-ALL); high hyperdiploid (HHD); diffuse large cell lymphoma (DLBCL); breast cancer (BC); small for gestational age (SGA); maternal grandmother (GM (M)); maternal grandfather (GF (M)); paternal grandmother (GM (F)); paternal grandfather (GF (F)); paternal uncle (U(F)); paternal great-grandfather GGP (F); maternal aunt (A (M)); grandfather's aunt (GFA); father’s cousin (C (F)); maternal uncle(U(M)); *in vitro* fertilization (IVF); Epstein-Barr Virus (EBV); Cytomegalovirus (CMV); years (y); atrial septum defect (ASD); rhabdomyosarcoma (RMS); hematopoietic stem cell transplantation (HSCT); Peutz-Jeghers syndrome (PJS). All LP/PVs were determined to be heterozygous, except for the *NBN* founder mutation in case LPP_12(ł), who was therefore excluded from monoallelic burden testing.

Supplementary Table 4. Details on determined (likely) pathogenic variants and pathogenicity classification.

LPP case	Finding and molecular consequence	Highest MAF[a]	Splice AI[b]	Bayes Del[b]	CADD[b]	REVEL[b]	Coverage	VAF	ACMG criteria met[c,d]	Variant classification reviewed/ curated by expert panel[d]	Final Classification[d]
01	*ATM*(ENST00000278616.8):c.5347_5350del p.(Glu1783Thrfs*9)	absent	0.16	-	-	-	77	0.455	PVS1, PM2, PM3_moderate	Not yet reviewed by expert panel; reported in a compound-heterozygous state in one case with AT (PMID: 33376610)[9]	P
02	*ATM*(ENST00000278616.8):c 3880dup p.(Ile1294Asnfs*8)	absent	0.07	-	-	-	215	0.433	PVS1, PM2, PM3_moderate	Not yet reviewed by expert panel; ClinVar 6 submissions: 1x LP,1xP (PMID: 21778326)[10]	P
03	*ATM*(ENST00000278616.8):c.1564_1565del p.(Glu522Ilefs*43)	absent	0	-	-	-	35	0.343	PVS1, PM3_very_strong	Not yet reviewed by expert panel; ClinVar: 10 submissions: 10x P	P
04	*ATM* (ENST00000278616.8):c.8766dup p.(Val2923Cysfs*2)	absent	0.01	-	-	-	190	0.516	PVS1, PM2, PM3_moderate	Not yet reviewed by expert panel; ClinVar: 4 submissions: 4xP	P
05	*TP53*(ENST00000269305.8):c.97-6_129del p.?	absent	-	-	35	-	166	0.373	PVS1, PM2	Not yet reviewed by expert panel; ClinVar 1 submission: 1xLP	LP
06	*TP53*(ENST00000269305.8):c.733G>A p.Gly245Ser	0.0000243677 (AFR)	0	0.55	27.5	0.947	66	0.515	PM1, PP3, PS4, PS3	ClinGen: Pathogenic	P
07	*TP53*(ENST00000269305.8):c.586C>T p.(Arg196*)	absent	0	0.66	39	-	82	0.171	PVS1, PM1, PM2	Not yet reviewed by expert panel; ClinVar 18 submissions: 18x P	P
08	*TP53*(ENST00000269305.8):c.537T>G p.His179Gln	absent	0	0.44	12.33	0.79	96	0.469	PS1, PS2, PS3, PM1, PM2, PP3	Not yet reviewed by expert panel; ClinVar 26 submissions: 23x LP, 3x P	P
09	*TP53*(ENST00000269305.8):c.919+1G>A p.?	absent	0.99	0.66	33	-	220	0.491	PM2_Supporting, PVS1_Strong, PM6_Supporting	ClinGen: Likely pathogenic	LP
10	*TP53*(ENST00000269305.8):c.586C>T p.(Arg196*)	absent	0	0.66	39	-	46	0.413	PVS1, PM1, PM2	Not yet reviewed by expert panel; ClinVar 18 submissions: 18x P	P
11	*NBN*(ENST00000265433.7):c.657_661del p.(Lys219fs)	absent	-	-	-	-	64	0.439	-	OMIM: known founder mutation (*NBN* allelic variant n°0001)	P
12	*NBN*(ENST00000265433.7):c.657_661del p.(Lys219fs)ǂ	absent	-	-	-	-	63	0.794	-	OMIM: known founder mutation (*NBN allelic variant n°0001)*	P
13	*NBN*(ENST00000265433.7):c.657_661del p.(Lys219fs)	absent	-	-	-	-	29	0.379	-	OMIM: known founder mutation (*NBN* allelic variant n°0001)	P
14	*BRIP1*(ENST00000259008.6):c.2992_2995del p.(Lys998Glu*60)	absent	-	-	-	-	210	0.329	PVS1, PM2	Not yet reviewed by expert panel; ClinVar: 14x P, 1x LP, 1x VUS; (PMID: 18628483)[11]	P(class 5[5])
15	*BRIP1*(ENST00000259008.6):c.2400C>G p. (Tyr800*)	0.0000154397 (NFE)	-	-	35	-	63	0.476	PVS1, PM2, PM3	Not yet reviewed by expert panel; (PMID: 28152038)[12]; ClinVar: 4 submissions: 3x P, 1x LP	P(class 5[5])
16	*MSH2*(ENST00000233146.6:):c.1386+1G>T p.?	absent	-	-	34	-	68	0.382	-	InSiGHT: Class 4: likely pathogenic (Interrupts canonical donor splice site)	LP
17	*CHEK2*(ENST00000382580.6):c.1031del p.(Leu344Trpfs*3)	absent	0	-	-	0	17	0.529	PVS1, PS4, (PM2)[6]	Not yet reviewed by expert panel; (absent protein expression and hence phosphorylation)[13]	P(class 5[5])

LPP case	Finding and molecular consequence	Highest MAF[a]	Splice AI[b]	Bayes Del[b]	CADD[b]	REVEL[b]	Coverage	VAF	ACMG criteria met[c,d]	Variant classification reviewed/ curated by expert panel[d]	Final Classification[d]
18	*CHEK2*(ENST00000382580.6):c.628G>A p.(Gly210Arg)	0.0000154297 (NFE)	0	0.57	26.8	0.955	132	0.561	PS3, PP1, PP3, (PM2)[6]	Not yet reviewed by expert panel; (decreased expression and hence phosphorylation)[13]	LP(class 4[5])
19	*CHEK2*(ENST00000382580.6):c.573+1G>A p.?	0.000169732 (NFE)	1.0	-0.08	34	0	173	0.399	PVS1, PS4, BS1	OMIM: *CHEK2* allelic variant n°0013	P(class 5[5])
20	*CHEK2*(ENST00000382580.6):c.1165C>T p.(Arg389Cys)	0.0000663922 (AMR)	0.11	0.27	32	0.78	151	0.44	PS3, PM1, PP3	Not yet reviewed by expert panel; (functional damaging)[14]	VUS(class 3)[5] > LP(class 4[5])
20	*NF1*(ENST00000358273.8):c.4137dup p.(Ala1380Serfs*15)	0.0000925812 (NFE)	-	-	-	-	151	0.44	PVS1, PM2	Not yet reviewed by expert panel; ClinVar: not found	LP
21	*CHEK2*(ENST00000382580.6):c.1165C>T p.(Arg389Cys)	0.0000663922 (AMR)	0.11	0.27	32	0.78	119	0.487	PS3, PM1, PP3	Not yet reviewed by expert panel; (functional damaging)[14]	VUS(class 3)[5] > LP
22	*NF1*(ENST00000358273.8):c.6819+3del p.?	absent	-	-	-	0	-	-	PS2, PM2, PP3, PP4	Not yet reviewed by expert panel	LP
23	*BRCA1*(ENST00000471181.7):c.5158C>T p.(Arg1720Trp)	absent	-	-	32	0.817	49	0.469	-	BRCA Exchange: Pathogenic	P
24	*FANCC*(ENST00000289081.8):c.37C>T p. (Gln13*)	absent	0.1	0.65	35	-	240	0.458	PVS1, PM2, PM3	Not yet reviewed by expert panel	P
25	*FANCM*(ENST00000267430.10):c.4064del p. (Lys1355Argfs*14)	absent	0	-	-	-	26	0.308	PVS1, PM2	Not yet reviewed by expert panel	LP
26	*BLM*(ENST00000355112.8):c.3558+1G>T p.?	absent	1	0.66	34	-	76	0.5	PVS1, PM2, BS1	Not yet reviewed by expert panel	LP
27	*STK11*(ENST00000326873.11):c179dup p.(Tyr60*)	absent	0	-	-	-	123	4.07	PVS1, PM2	Not yet reviewed by expert panel	LP

Abbreviations: Patient identifier for carrier of a (likely) pathogenic variant (LPP); acute lymphoblastic leukemia (ALL); second malignant neoplasm (SMN); single nucleotide variants (SNV); minor allele frequency (MAF); variant allele fraction (VAF).

[a] Highest MAF observed in one of the recommended large subpopulations of gnomAD (v.3.1.2; non-cancer dataset): non-Finnish European (NFE), African/African American (AFR), Admixed American (AMR); East-Asian (EAS) or South-Asian (SAS).

[b] The following in silico prediction scores available from e.g. the gnomAD and or ensembl websites, were considered for ACMG/AMP the PP3/BP1 rules, to assess the impact of the variant on gene function and splicing: The REVEL metascore is an ensembl method for predicting the pathogenicity of missense variants based on a combination of scores from 13 individual tools: MutPred, FATHMM v2.3, VEST 3.0, PolyPhen-2, SIFT, PROVEAN, MutationAssessor, MutationTaster, LRT, GERP++, SiPhy, phyloP, and phastCons[15] and levels >0.75 indicate a pathogenic effect. SpliceAI uses deep neural networks to predict splicing events. The score can range from 0 to 1, where scores can be interpreted as the probability of the variant being splice-altering; BayesDel (no AF)[16] is a deleteriousness meta-score, ranging from -1.29334 to 0.75731. The higher the score, the more likely the variant is pathogenic. In addition, the Combined Annotation Dependent Depletion (CADD) score was used to interpret the deleteriousness of single nucleotide and insertion/deletion variants [17] (https://cadd.gs.washington.edu/).

[c] ACMG/AMP criteria[3] met by the respective variant. Classifications according to non-ACMG-based specifications like Wappenschmidt *et al.*[5] (e.g. for *BRIP1*, *RAD51C*, *RAD51D*, *CHEK2*) are noted in the last column and considered for the final classification.

[d] All variants were evaluated independently by two investigators. The final conclusion/classification regarding the ACMG criteria and/or specific refinements is given here as likely pathogenic (LP) or pathogenic (P). Expert panel reviews were considered when available in the databases of ClinGen, InSiGHT, BRCA Exchange or OMIM (individual links are provided above). Variant-related ClinVar submissions were also listed above, when no curated expert panel classification was available. All LP/PVs were determined to be heterozygous, except for the *NBN* founder mutation in case LPP_12(ɫ), who was therefore excluded from monoallelic burden testing.

Supplementary Table 5. Information on 187 determined variants of uncertain significance (VUS) in 372 analyzed pediatric cancer patients. (see additional supplementary excel file).

Supplementary Table 6. Joint burden testing results by gene.

Gene[a]	healthy adults +LP/PV[b] (n=74023)	healthy adults -LP/PV[b] (n=74023)	pediatric cases +LP/PV[c](n=1492)	pediatric cases -LP/PV[c](n=1492)	OR (95% CI)[d]	*P*[d]	LOEUF[e]
TP53[f]	37	73986	56	1436	78.0 (51.3-118.5)	$7.68x10^{-71}$	0.469
NF1[f]	41	73982	6	1486	7.3 (3.1-17.2)	$3.16x10^{-4}$	0.285
NBN	43	73980	3	1488	3.5 (1.1-11.2)	0.0623	1.010
MSH2	14	74009	2	1490	7.1 (1.6-31.2)	0.0390	0.334
CHEK2	105	73918	9	1483	4.3 (2.2-8.5)	$4.55x10^{-4}$	1.522
STK11[f]	1	74022	1	1491	49.6 (3.1-794.1)	0.0391	0.245
FANCM	91	73932	3	1489	1.6 (0.5-5.2)	0.4374	0.593
FANCC	151	73872	2	1490	0.7 (0.2-2.7)	0.7735	1.043
BRIP1	95	73928	3	1489	1.6 (0.5-5)	0.4472	0.786
BRCA1	103	73920	2	1490	1.0 (0.2-3.9)	1.0000	0.920
BLM[f]	118	73905	2	1490	0.8 (0.2-3.4)	1.0000	0.751
ATM	251	73772	7	1485	1.4 (0.7-2.9)	0.3644	0.710

Abbreviations: (likely) pathogenic variants (LP/PVs); heritable breast and ovarian cancer (HBOC); odds ratio (OR); 95% confidence intervals (95% CI); p-value (*P)*; pathogenic loss of function (pLoF).

[a] Approved gene symbols, according to the human gene nomenclature committee (HGNC) are given.

[b] We included all available healthy adults from the Genome Aggregation Database (gnomAD) non-cancer set version 3.1.1 as controls (healthy adults, n=74023) in this subsequent joint burden testing.

[c] For this second approach we combined the number of LP/PVs per gene and patient in our study cohort (n=372) with previously published data on pediatric cancer patients (Zhang *et al* 2015, n=1120)[18]; we compared the data of this joint pediatric cohort (n_total=1492) with the frequencies in the healthy adult control population. As in our initial approach only individuals with monoallelic / heterozygous LP/PVs were included; and again for *NBN* one individual with a homozygous *NBN* variant was excluded from our internal cohort (for details see Suppl. table 1).

[d] For burden analyses we performed logistic regression analyses plus 2-sided Fisher's exact tests for each gene. Results were considered to be relevant for p-values <0.05. Significant associations were determined for: *TP53*, *NF1*, *MSH2, CHEK2* and *STK11*; results for *STK11* should be interpreted carefully, due to the overall rarity of LP/PVs in this gene (for a graphical summary see Suppl. Figure 3).

[e] As a metric for evolutionary pressure, mutational constraint scores are added to indicate the tolerance of a gene regarding loss off function variation in the normal (adult) population[19,20]: i.e. upper bound of 90% confidence interval for observed vs. expected ratio for pLoF variants (LOEUF/oe_lof_upper, gnomAD v.2.1.1); genes with LOEUF scores <0<0.35 are considered to be constrained.

[f] Diseases related to these genes exclusively follow an autosomal dominant inheritance mode.

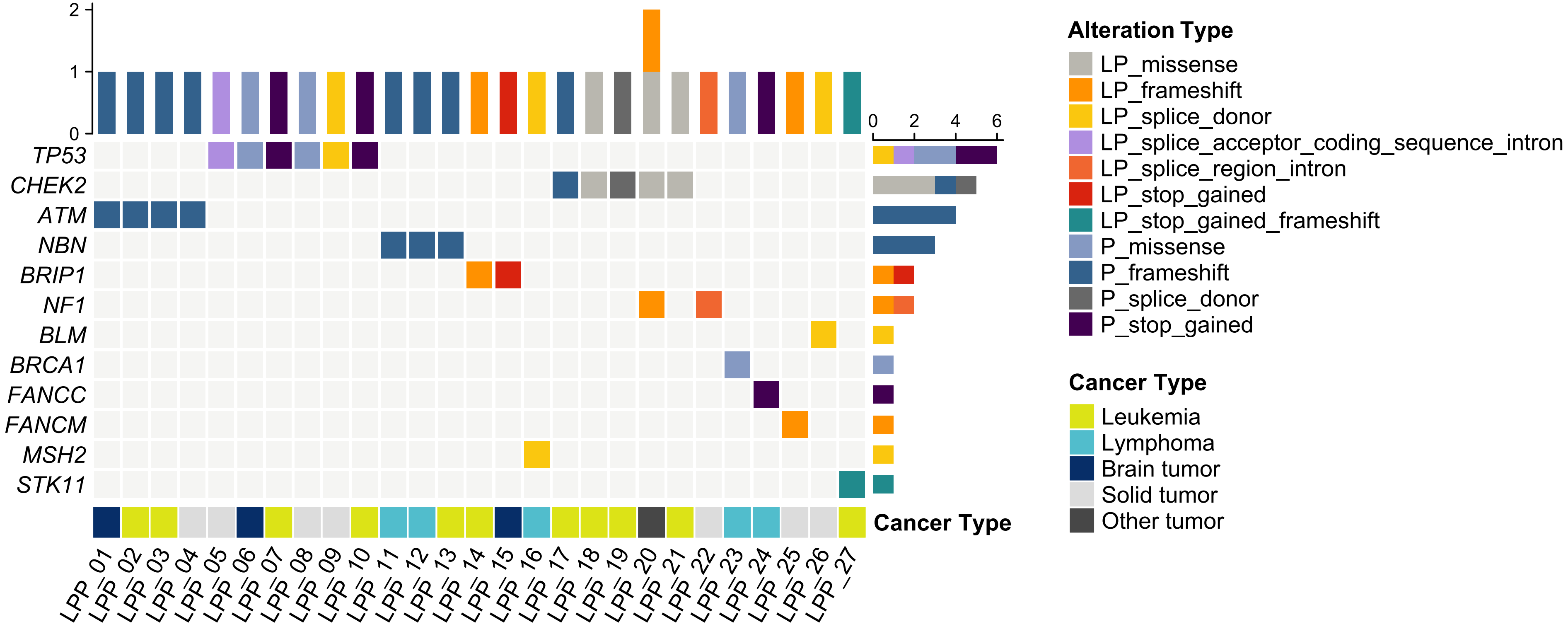

Supplementary Figure 1. Oncoprint visualization of all 28 determined (likely) pathogenic (LP/P) variants by gene and the categorized first cancer entity of the carrier. As explained above, the colors reflect the molecular consequence ("Alteration Type") of the variant in the upper part and in the lower part the first cancer entity category ("Cancer Type") of the affected 27 pediatric patients (LPP_01 to LPP_27); the numbering corresponds to the patient identifier applied in Suppl. Tables 2 to 5.

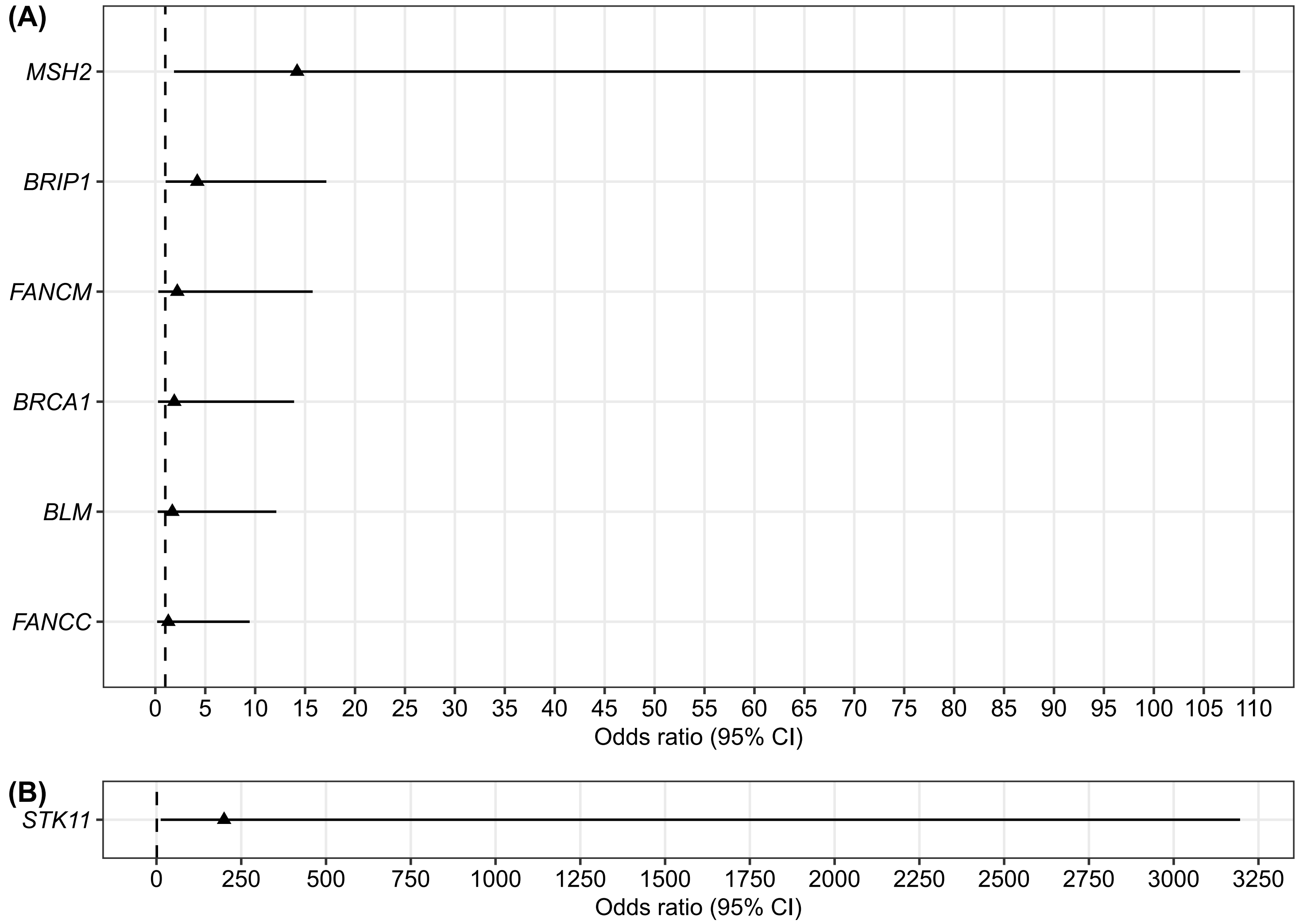

Supplementary Figure 2. Non-significant burden test results by gene. (A) Genes in which only one individual out of 372 in our prospective single-institution cohort of pediatric cancer patients carried a (likely) pathogenic variant were not considered to be of statistical importance when compared to the normal non-cancer population data set available from the Genome Aggregation Database (gnomAD non-cancer,3.1.1, n=74023); see Table 2 for further details.
(B) Due to the large 95% confidence interval *STK11* was plotted in a separate plot panel.

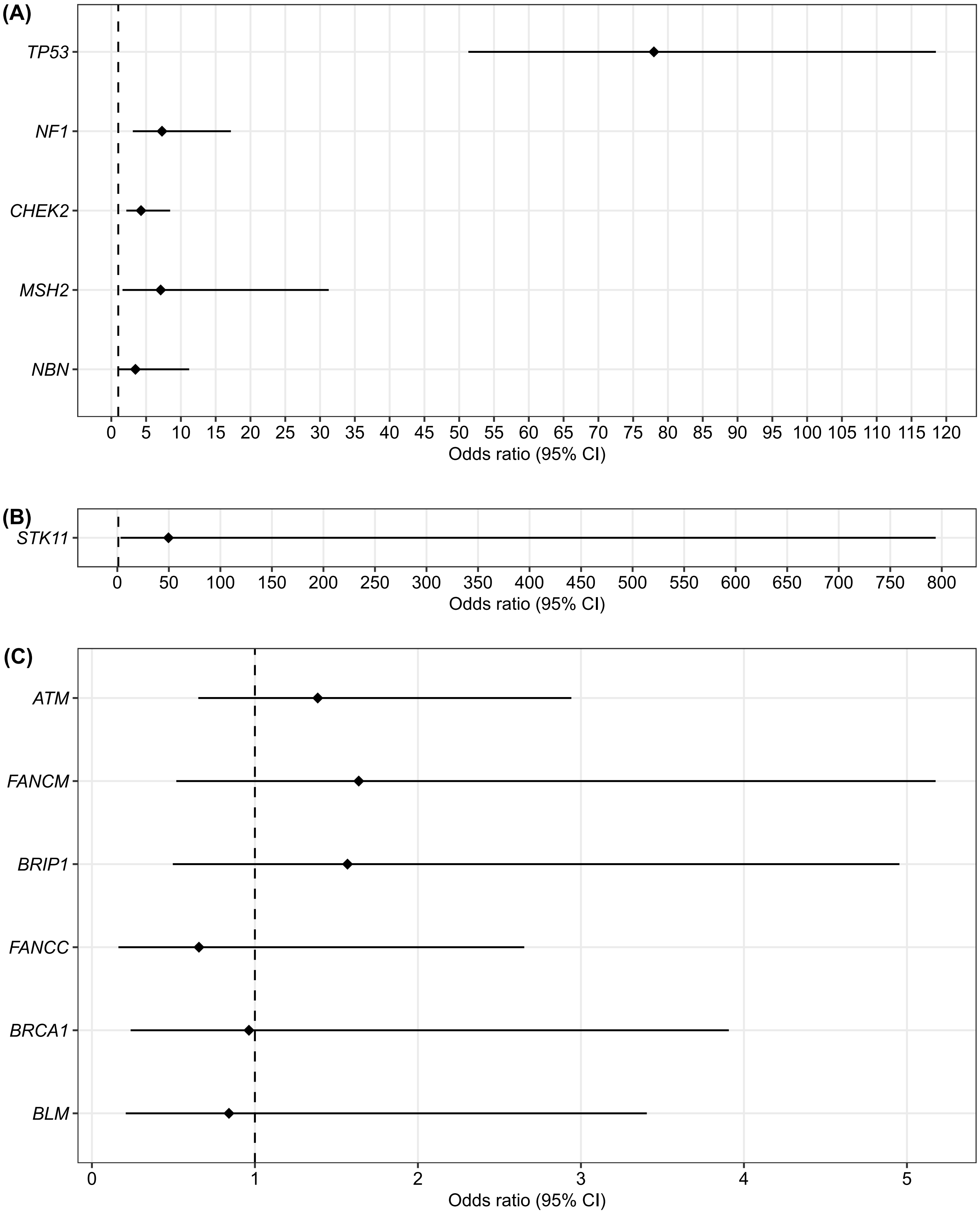

Supplementary Figure 3. Joint burden test results by gene. Number of monoallelic (likely) pathogenic variants in 1492 pediatric cancer patients compared to the normal non-cancer population data set available from the Genome Aggregation Database (gnomAD non-cancer, 3.1.1) (A) significant associations were obtained for *TP53*, *NF1*, *NBN*, *MSH2* and *CHEK2*; results that require additional validation were obtained for (B) *STK11* and (C) *FANCM*, *FANCC*, *BRIP1*, *BRCA1*, *BLM* and *ATM*. A statistical summary can be found in Suppl. Table 6.

Supplementary References

1. Brozou T, Taeubner J, Velleuer E, et al. Genetic predisposition in children with cancer - affected families' acceptance of Trio-WES. *Eur J Pediatr*. Jan 2018;177(1):53-60. doi:10.1007/s00431-017-2997-6

2. McLaren W, Gil L, Hunt SE, et al. The Ensembl Variant Effect Predictor. *Genome Biology*. 2016/06/06 2016;17(1):122. doi:10.1186/s13059-016-0974-4

3. Richards S, Aziz N, Bale S, et al. Standards and guidelines for the interpretation of sequence variants: a joint consensus recommendation of the American College of Medical Genetics and Genomics and the Association for Molecular Pathology. *Genet Med*. May 2015;17(5):405-24. doi:10.1038/gim.2015.30

4. Rehm HL, Berg JS, Brooks LD, et al. ClinGen — The Clinical Genome Resource. *New England Journal of Medicine*. 2015;372(23):2235-2242. doi:doi:10.1056/NEJMsr1406261

5. Wappenschmidt B, Hauke J, Faust U, et al. Criteria of the German Consortium for Hereditary Breast and Ovarian Cancer for the Classification of Germline Sequence Variants in Risk Genes for Hereditary Breast and Ovarian Cancer. *Geburtshilfe und Frauenheilkunde*. Apr 2020;80(4):410-429. doi:10.1055/a-1110-0909

6. Young CC, Feng B-J, Mackenzie CB, et al. Evaluation of ACMG Rules for In silico Evidence Strength Using An Independent Computational Tool Absent of Circularities on *ATM* and *CHEK2* Breast Cancer Cases and Controls. *bioRxiv*. 2019:835264. doi:10.1101/835264

7. Fortuno C, Richardson M, Pesaran T, et al. CHEK2 is not a Li-Fraumeni syndrome gene: time to update public resources. *J Med Genet*. Nov 27 2023;60(12):1215-1217. doi:10.1136/jmg-2023-109464

8. Thompson BA, Spurdle AB, Plazzer JP, et al. Application of a 5-tiered scheme for standardized classification of 2,360 unique mismatch repair gene variants in the InSiGHT locus-specific database. *Nat Genet*. Feb 2014;46(2):107-115. doi:10.1038/ng.2854

9. Hettiarachchi D, Panchal H, Pathirana B, et al. Six Novel ATM Gene Variants in Sri Lankan Patients with Ataxia Telangiectasia. *Case Rep Genet*. 2020;2020:6630300. doi:10.1155/2020/6630300

10. Keimling M, Volcic M, Csernok A, Wieland B, Dork T, Wiesmuller L. Functional characterization connects individual patient mutations in ataxia telangiectasia mutated (ATM) with dysfunction of specific DNA double-strand break-repair signaling pathways. *FASEB J*. Nov 2011;25(11):3849-60. doi:10.1096/fj.11-185546

11. De Nicolo A, Tancredi M, Lombardi G, et al. A novel breast cancer-associated BRIP1 (FANCJ/BACH1) germ-line mutation impairs protein stability and function. *Clin Cancer Res*. Jul 15 2008;14(14):4672-80. doi:10.1158/1078-0432.Ccr-08-0087

12. LaDuca H, Farwell KD, Vuong H, et al. Exome sequencing covers >98% of mutations identified on targeted next generation sequencing panels. *PLoS One*. 2017;12(2):e0170843. doi:10.1371/journal.pone.0170843

13. Wagener R, Walter C, Auer F, et al. The CHK2 kinase is recurrently mutated and functionally impaired in the germline of pediatric cancer patients. *International Journal of Cancer*. 2023;152(7):1388-1398. doi:https://doi.org/10.1002/ijc.34390

14. Delimitsou A, Fostira F, Kalfakakou D, et al. Functional characterization of CHEK2 variants in a Saccharomyces cerevisiae system. *Human Mutation*. 2019;40(5):631-648. doi:https://doi.org/10.1002/humu.23728

15. Ioannidis NM, Rothstein JH, Pejaver V, et al. REVEL: An Ensemble Method for Predicting the Pathogenicity of Rare Missense Variants. *Am J Hum Genet*. Oct 6 2016;99(4):877-885. doi:10.1016/j.ajhg.2016.08.016

16. Pejaver V, Byrne AB, Feng BJ, et al. Calibration of computational tools for missense variant pathogenicity classification and ClinGen recommendations for PP3/BP4 criteria. *Am J Hum Genet*. Dec 1 2022;109(12):2163-2177. doi:10.1016/j.ajhg.2022.10.013

17. Rentzsch P, Schubach M, Shendure J, Kircher M. CADD-Splice—improving genome-wide variant effect prediction using deep learning-derived splice scores. *Genome Medicine*. 2021/02/22 2021;13(1):31. doi:10.1186/s13073-021-00835-9

18. Zhang J, Walsh MF, Wu G, et al. Germline Mutations in Predisposition Genes in Pediatric Cancer. *N Engl J Med*. Dec 10 2015;373(24):2336-46. doi:10.1056/NEJMoa1508054

19. Stoltze UK, Foss-Skiftesvik J, Hansen TVO, et al. The evolutionary impact of childhood cancer on the human gene pool. *Nat Commun*. Feb 29 2024;15(1):1881. doi:10.1038/s41467-024-45975-9

20. Karczewski KJ, Francioli LC, Tiao G, et al. The mutational constraint spectrum quantified from variation in 141,456 humans. *Nature*. May 2020;581(7809):434-443. doi:10.1038/s41586-020-2308-7
